# Comorbidities in combined retinal artery and vein occlusions

**DOI:** 10.1186/2047-783X-18-27

**Published:** 2013-08-16

**Authors:** Dieter Schmidt

**Affiliations:** 1 University-Augenklinik, Killianstr. 5, Freiburg D-79106, Germany

## Abstract

**Background:**

Several general diseases cause blindness in patients with simultaneous combined retinal artery and vein occlusion.

**Methods/patients:**

We examined 14 patients with acute unilateral visual loss due to combined retinal artery and venous occlusions. All 14 patients presented at the Polyclinic over a period of about 3 years. Fluorescein angiography was carried out in 12 patients to confirm the diagnosis. Ten patients underwent Doppler sonography and 11 echocardiography.

**Results:**

Concerning systemic diseases, 11 of our 14 patients presented several cardiovascular risk factors, i.e., immunocytoma and arterial hypertension and hypercholesterolemia in one patient; another patient had chronic bronchitis, tachycardia and hypercholesterolemia. Six patients presented coagulation anomalies, and eight patients had arterial hypertension.

Doppler sonography revealed normal carotid arteries in nine of ten patients. In 8 of 11 patients, echocardiography displayed no cardiac abnormalities.

Ophthalmoscopy revealed no emboli in any of these patients.

**Conclusion:**

Unilateral simultaneous combined incomplete retinal artery and venous occlusions should be considered as one entity. Eleven of our patients presented comorbidities reflecting several cardiovascular risk factors. Immunological diseases, malignancies and coagulopathies can cause this ocular disorder, resulting in blindness. No emboli were found in any of these patients. Patients suffering from acute visual loss must be examined for the presence of systemic diseases to enable therapy at an early stage.

## Background

Systemic diseases in noninflammatory isolated branch or central retinal artery occlusions or isolated branch or central retinal vein occlusions have been described in large patient cohorts by several authors [1-3]. Regarding the rare entity of combined retinal artery and venous occlusions, cardiovascular risk factors have generally been documented in case reports involving few patients in most publications [4-50].

Three different types of combined retinal artery and vein occlusions (often incomplete) have been reported:

1. Central retinal vein occlusion (CRVO) with central retinal artery occlusion (CRAO).

2. CRVO with branch retinal artery occlusion (BRAO).

3. CRVO with cilioretinal artery occlusion (CLRAO).

Criteria for the entity of combined retinal artery and venous occlusion are acute unilateral visual loss with retinal edema (retinal whitening of the posterior pole or along an artery), with or without a cherry red spot on the macula, retinal hemorrhages, enlarged, tortuous veins, delayed arterial dye filling of arteries and prolonged arteriovenous transit time in fluorescein angiography. Complete occlusions of the central retinal vein and central retinal artery are extremely rare. Several publications have reported central retinal vein occlusion in association with cilioretinal artery occlusion [7,22,23,29-32,41,42,48]. Hayreh et al. [22] argued that sudden CRVO associated with cilioretinal artery occlusion results in a marked rise in intraluminal pressure in the capillary bed. Cilioretinal artery occlusion is a hemodynamic block, not caused by emboli.

McLeod [31] pointed out that the probability of cilioretinal infarction increases with the increasing severity of CRVO. Pathogenesis of the combined CRVO with CLRAO is regarded as a particular entity that differs from the pathogenesis of combined central retinal artery obstruction and central retinal vein occlusion.

The choroidal arterial steal mechanism can influence the development of combined CRV and CRA occlusions.

Unilateral combined occlusion of the central retinal artery (CRAO) and central retinal vein (CRVO) was first described by Gross in 1907 [18] in a 48-year-old woman with anemia. Different causes of combined central retinal artery and vein occlusion have been reported. We propose various explanations for the disease’s development in our 14 patients.

## Case presentation

### Methods and patients

The 14 patients (5 female and 9 male) had a mean age of 57.6 years (33–75). All were examined at the Polyclinic of the University Eye Hospital over a period of approximately 3 years. The right eye was affected in nine, the left eye in five patients. The patients were referred because of acute unilateral visual loss.

**Figure 1:**
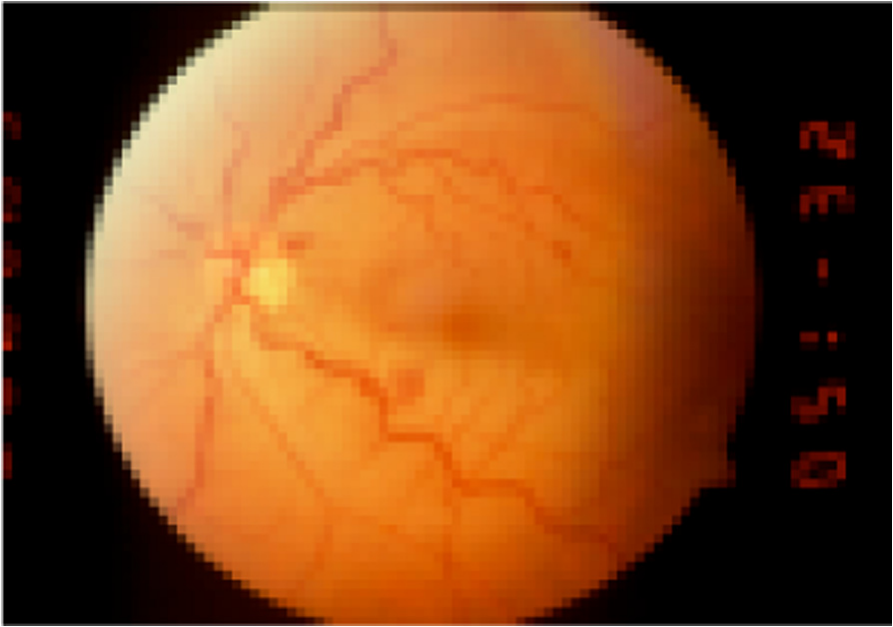
LE: Left Eye Initial stage of infarction of the inferior temporal artery associated with a few dot-and-blot hemorrhages and enlarged, tortuous veins in the left eye of a 62-year-old man (patient no. 4).

**Figure 2:**
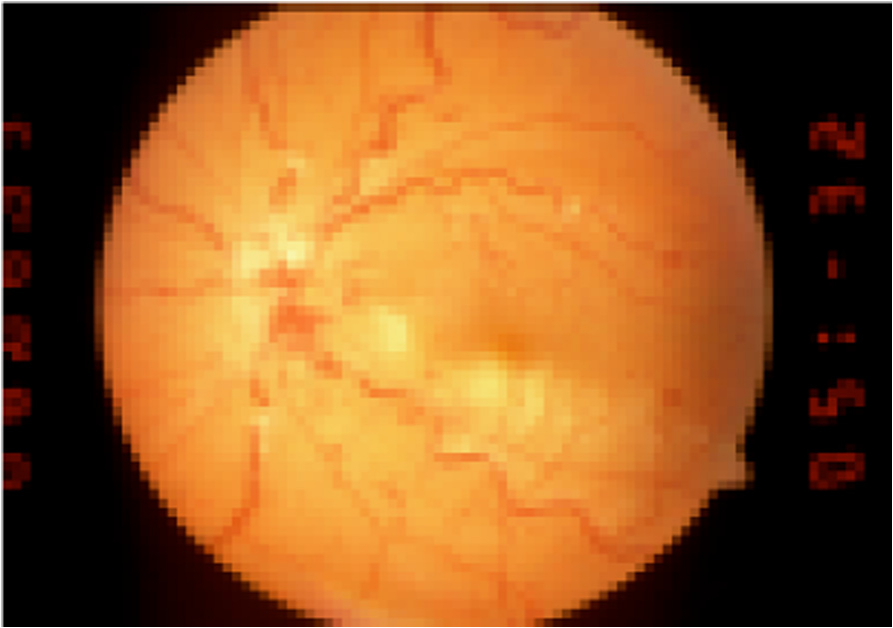
A new episode with pronounced retinal changes: papilledema, cotton-wool spots and additional hemorrhages (2 weeks later with deterioration of retinal findings, patient no. 4).

**Figure 3:**
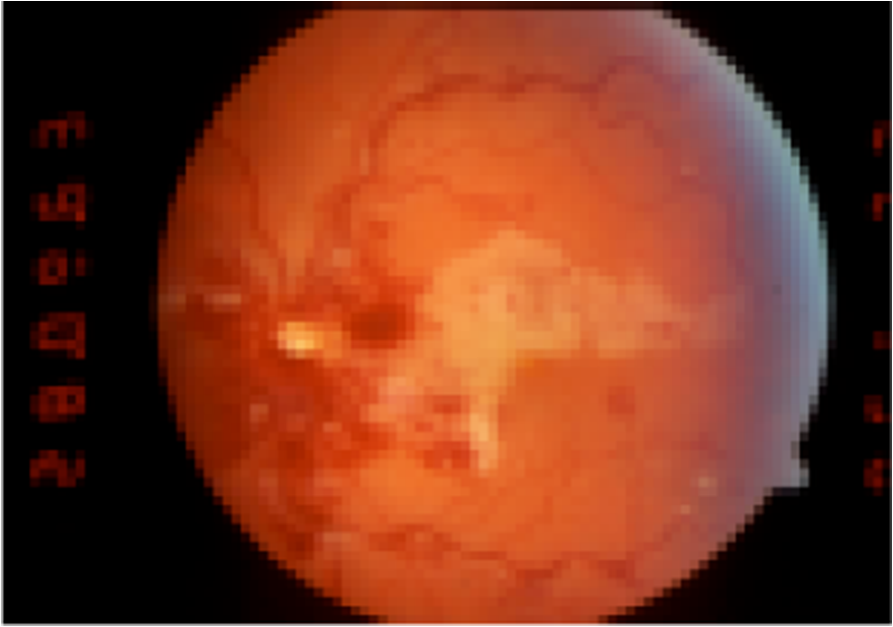
LE: Infarction of a large cilioretinal artery associated with a CRVO in the left eye of a 39-year-old male who had arterial hypertension (patient no. 14).

**Figure 4:**
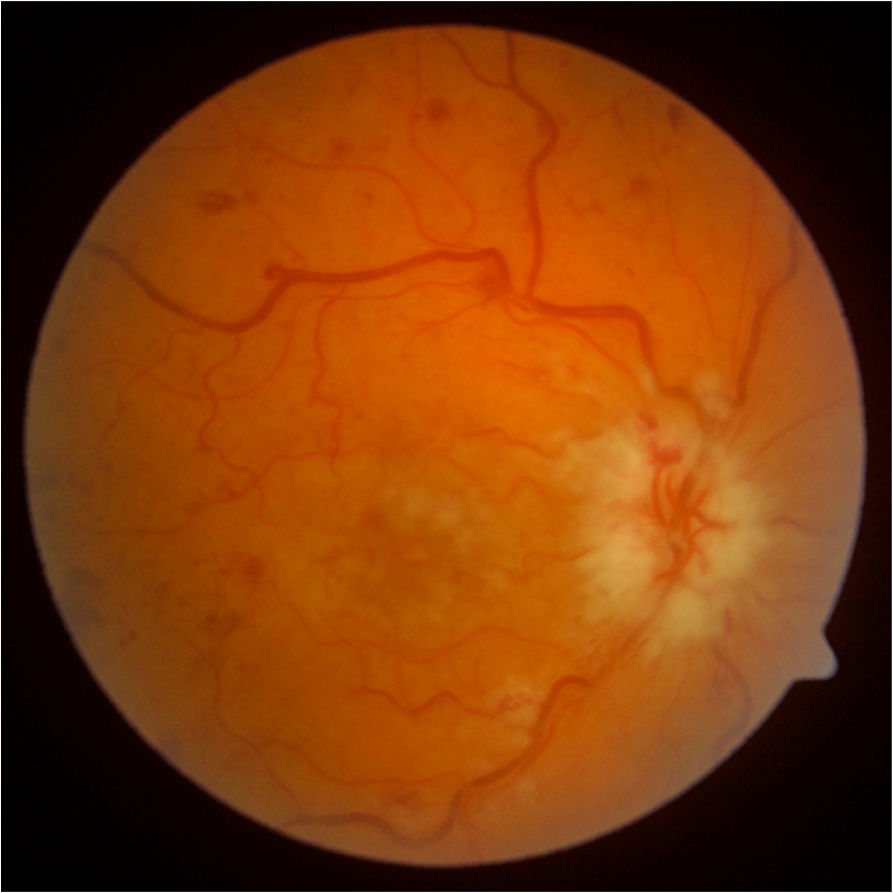
**RE: Right Eye in a 57-year-old woman.** Swelling of the optic disk, macular edema, macular cherry-red spot and multiple retinal hemorrhages (patient no. 10).

**Figure 5:**
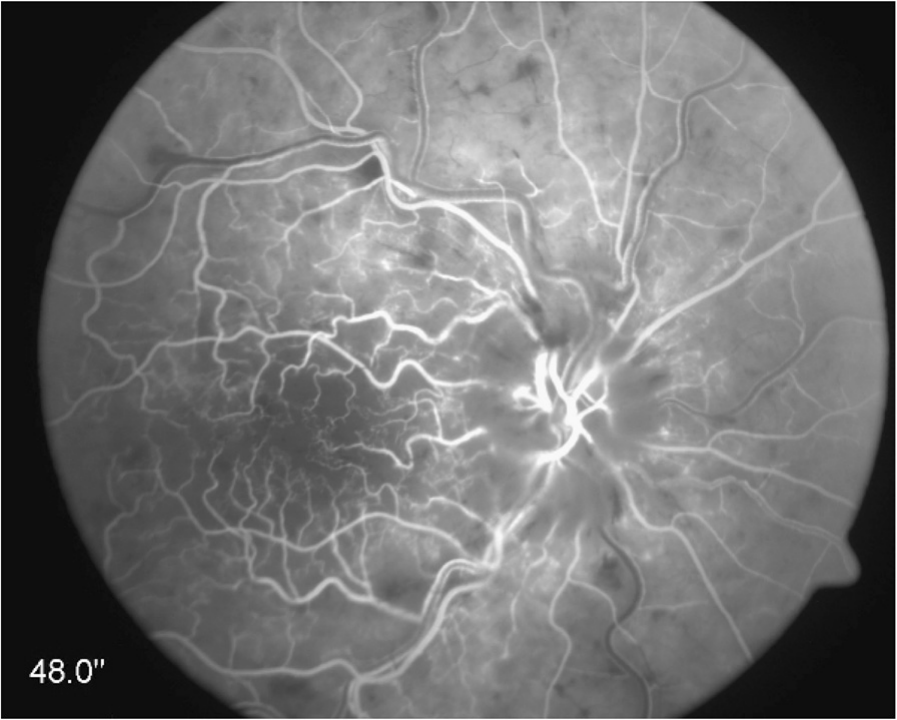
Fluorescein-angiogram: 48 s after dye injection: delayed filling of vessels, tortuosity of vessels mainly in the area of the posterior pole, some peripheral vessels are not filled with fluorescein (temporal superior and nasal inferior areas) (patient no. 10).

**Figure 6:**
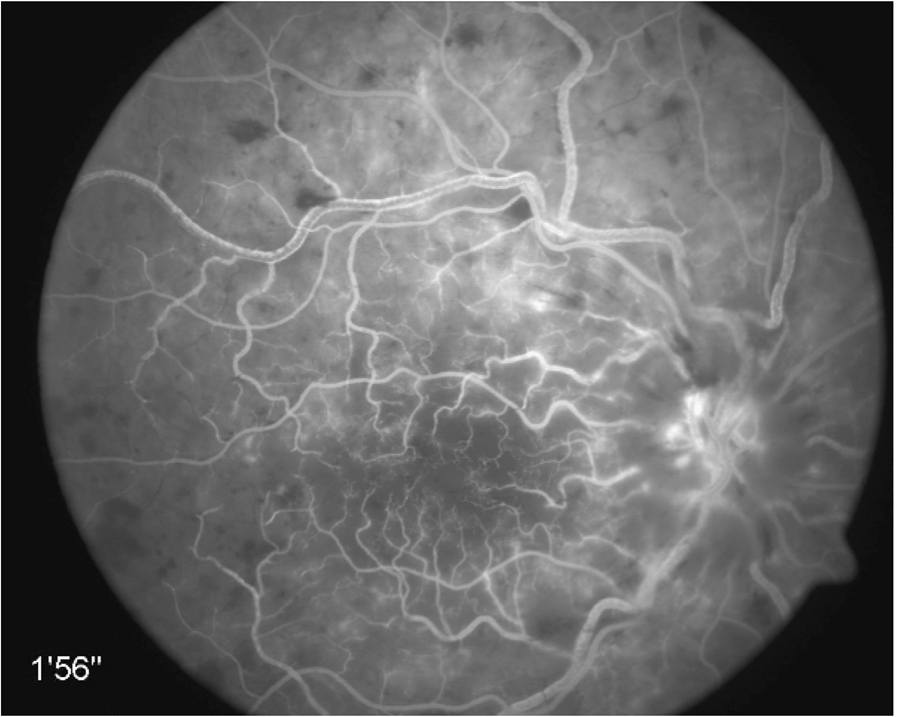
**Fluorescein-angiogram: 1 min, 56 s after dye injection.** All vessels are filled with dye. Sludge phenomenon in superior temporal veins (patient no. 10).

Unilateral combined retinal artery and vein occlusions were observed in all our patients. Ten (Table 1) presented combined CRVO and CRAO, and three patients had combined CRVO and BRAO (Table 2). One patient showed a combined cilioretinal artery occlusion (CLRAO) and CRVO Figures 1, 2, 3, 4, 5, 6.

**Table 1:** Case histories of ten patients with combined CRVO and CRAO

**Age of patients**	**Diagnoses of systemic diseases (number of patients)**
1. A 75-year-old male	Immunocytoma (Waldenström’s disease); arterial hypertension (200/130 mmHg); hypercholesterolemia, adiposity
2. A 61-year-old male	Pulmonary emphysema, cor pulmonale, tachycardia; + chronic bronchitis; hypercholesterolemia heart-echo: normal Doppler sonography: normal carotid arteries
3. A 70-year-old woman	Arterial hypertension (200/100 mmHg); hypercholesterolemia, heart-echo: normal findings Doppler sonography: normal carotid arteries
4. A 62-year-old male	Slightly increased blood pressure, slightly increased homocysteine, heart-echo: normal findings Doppler sonography: normal findings in carotid arteries
5. A 36-year-old woman	Raynaud’s disease; autoimmune disease with evidence of serum antimitochondrial antibodies, tendency for arterial hypotension with occasional low blood pressure (90/60 mmHg); heart-echo normal findings Doppler sonography: normal carotid arteries
6. A 68-year-old male	Metastatic prostate carcinoma, suspected paraneoplastic syndrome with hypercoagulation of blood; anemia (hemoglobin. 6.7 g/dl), diabetes mellitus; elevated ESR 74 mm (Westergren method); heart-echo: normal findings Doppler sonography: normal carotid arteries
7. A 53-year-old male	Hyperhomocysteinemia, hyperuricemia, heart-echo: suspected diastolic relaxation disturbance, Parkinson’s disease, tendency to arterial hypotension, Doppler sonography: normal carotid arteries
8. A 72-year-old woman	Bronchopneumonia, leukocytosis, severely elevated ESR. Doppler sonography: bilateral medium-sized carotid stenoses
9. A 68-year-old male	Heterozygote factor-V mutation, venous thrombosis of the legs, coronary heart disease, arterial hypertension, small colon cancer, compensated renal insufficiency; heart-echo: normal findings
10. A 57-year-old woman	Factor V Leiden mutation (heterozygotic); arterial hypertension, chronic smoker; recurrent thrombosis of the lower right leg, Doppler-sonography: normal
Mean age was of the 4 women and 6 men was 62 years (36–75 yeras)	**Coagulation disturbances:** patients 4, 6, 7, 9, 10: 5 patients
	**Arterial hypertension:** patients. 1, 3, 4, 5, 9, 10: 6 patients
	**Arterial hypotension tendency:** patients 5, 7: 2 patients
	**Heart-echo:** normal findings patients 2, 3, 4, 5, 6, 9: 6 patients. Suspected diastolic relaxation disturbance: patient 7: 1 patient
	**Doppler sonography:** normal carotid artery findings patients: 2, 3, 4, 5, 6, 7, 10: 7 patients; 1 patient (no. 8) had bilateral medium-sized carotid stenoses

Abbreviations: *CRVO* central retinal vein occlusion, *CRAO* central retinal artery occlusion, *BRAO* branch retinal artery occlusion, CWSs cotton-wool spots, *RE* right eye, *LE* left eye, *Heart echo* echocardiography, *NLP* no light perception.

**Table 2:** Three patients with combined CRVO and BRAO

**Patient age**	**Diagnoses of systemic diseases**
	**(no. of patients)**
11. A 64-year-old male	Arterial hypertension (230/140 mmHg)
	Heart echo: ventricular hypertrophy
12. A 48-year-old male	Chronic smoker;
patient noticed an amaurosis fugax attack 2 weeks earlier	Heart-echo:
	Patent foramen ovale; arterial hypertension;
	Doppler sonography: normal carotid arteries
13. A 33-year-old woman	Taking hormones to become pregnant (Clonifen Galen, woman 1 Tbl/day) for 8 months; chronic smoker; coagulation disturbance due to increased factor VIII activity; heart-echo: normal findings. Cerebral MRT: normal findings
**Mean age: 2 males and 1 female: 48.3 (33–64 years)**	**Arterial hypertension:** patient 11
	**Chronic smoker**: patients 12, 13
	**Hormone treatment:** patient 13
	**Heart-echo:** normal findings: patient 13
	**Coagulation disturbance:** patient 13
	**Patent foramen ovale:** patient 12
	**Ventricular hypertrophy:** patient 11
	**Doppler sonography:** normal carotid arteries: patient 12

### Etiologies

Diagnoses of ocular and systemic diseases (including patients with cilioretinal artery occlusion and CRVO)

Ocular diseases

Posterior scleritis: (Shukla et al. [43]), blunt ocular trauma (Noble & Alvarez [34]), vitrectomy (Verma et al., [50]).

Retrobulbar anesthesia (Brown et al. [8], Giuffré et al. [16], Sullivan et al. [46]), thrombosis of the orbital superior ophthalmic vein (Hsu et al. [21]), meningeal carcinomatosis with compression of the retrobulbar optic nerve (Schaible & Golnik [40]).

Inflammatory ocular and/or orbital diseases of unknown origin (Bender [6]), papillophlebitis (Cassen et al. [9]) orbital inflammatory pseudotumor (Foroozan: [15]).

General diseases, immunological diseases.

Antiphospholipid syndrome (Ang et al. [4], Durukan [14]).

Systemic lupus erythematosus (Brown et al. [8], Cobo-Soriano et al. [11], Coppeto & Lessell [12]).

Churg-Strauss syndrome (Hamann [20]), Behçet’s disease (Richards [37]), interferon treatment (Jenisch et al. [24]).

Vascular diseases.

Arterial hypertension (Brazitikos et al. [7], Brown et al. [8], Limaye et al. [29], Vallé et al. [49], McLeod & Ring [30]), diabetes mellitus (Arikan et al. [5], Brown et al. [8]).

Migraine (Cassen et al. [9], Glacet-Bernard et al. [17]), Raynaud’s syndrome (Glacet-Bernard et al. [17]), smoker (Vallé et al. [49]).

Oral contraceptives (Brazitikos et al. [7], Glacet-Bernard et al. [17]), syphilis (Smith [44]).

Coagulopathy.

Activated protein C resistance (Vallé et al. [49]).

Inflammatory disease.

Sinus thrombosis.

Sinusitis and thrombosis in the sinus cavernosus (Richards: [37]).

Malignancies.

Prostate cancer (Jorizzo: [25]).

Non-Hodgkin’s lymphoma (Guyer et al. [19], Saatci [39]).

Lymphocytic leukemia (Chan et al. [10], Richards [37]).

In the ten patients with visual loss (Table 1), the right eye was affected in six and the left eye in four. For the examinations, fluorescein angiography was carried out in eight patients, revealing delayed arterial filling in six (patients 1, 2, 3, 4, 5, 10) and no filling delay in two (patients 7, 8). Optic disk swelling was observed in five patients (nos. 2, 3, 4, 8, 10); cotton-wool spots (CWSs) were detected in two patients (nos. 2 and 6).

Five patients (nos. 2, 3, 5, 7, 8) experienced distinct visual improvement after treatment, one patient (no. 6) a slight improvement, three patients (nos. 1, 9, 10) no visual change and one patient (no. 4) a slight deterioration.

In the three patients with visual loss (Table 2), the right eye was affected (nos. 11, 12, 13). All three underwent fluorescein-angiography, which revealed delayed arterial filling in one patient. We observed distinct visual improvement after treatment in one patient (no. 12) and no change in visual acuity in two patients (nos. 11 and 13).

In one patient with CLRAO and CRVO (no. 14), the left eye was affected. Fluorescein angiography revealed delayed dye filling of the cilioretinal artery. His visual acuity improved after treatment.

Doppler sonography was carried out in ten patients; nine had normal carotid arteries. Only one patient demonstrated bilateral, moderately sized carotid stenoses.

Echocardiography was carried out in 11 patients, yielding normal findings in 8 (nos. 2, 3, 4, 5, 6, 9, 13, 14). One patient (no. 7) was suspected of having a diastolic disturbance during myocardial relaxation, while another patient (no. 11) presented ventricular hypertrophy. Another patient (no. 12) revealed a patent foramen ovale. The 48-year-old male with the patent foramen ovale had arterial hypertension and was a chronic smoker (as co-morbidity).

Treatment in some patients consisted of isovolemic hemodilution, aspirin administration or other anticoagulants. One patient received corticosteroids (no. 1). In those with systemic diseases, treatment was carried out in cooperation with internists. In one patient (no. 1, Table 1) with combined CRVO and CRAO, vitrectomy and endolaser treatment of the retina were carried out. One patient (no. 14, Table 3) revealed a combined CRVO with a cilioretinal arterial occlusion (CLRAO). This 39-year-old male was diagnosed with increased diastolic blood pressure. His visual acuity was 20/400 initially, improving slightly to 20/200 after anticoagulant treatment and lowering his blood pressure.

**Table 3:** A patient with combined cilioretinal artery occlusion (CLRAO) and CRVO

**Patient age**	**Diagnoses of systemic diseases**
	**(no. of patients)**
14. A 39-year-old male	Arterial hypertension;
	**Doppler sonography:** normal carotid arteries
	**Heart echo:** normal findings

After treatment, we noted a distinct improvement in visual acuity (more than two lines) in six patients (nos. 2, 3, 5, 7, 8, 12), slight improvement in one patient (no. 6), no change in visual acuity in five patients (nos. 1, 9, 10, 11, 13, 14) and deterioration in one patient after a recurrence (no. 4).

The patients whose visual acuity improved substantially were those with BRAO and incomplete CRVO, autoimmune disease (no. 5), hyperhomocysteinemia (no. 7), hypertension (nos. 3, 12) and bronchopneumonia (nos. 2, 8).

## Results

Eight patients were diagnosed with arterial hypertension, three with hypercholesterolemia and six with coagulation anomalies. Three patients were chronic smokers. Eleven of our 14 patients presented comorbidities in the form of several cardiovascular risk factors.

Comorbidities were striking in our patients, as at least 11 of them presented several diseases, i.e., immunocytoma and arterial hypertension in conjunction with hypercholesterolemia (patient 1); chronic bronchitis with tachycardia and hypercholesterolemia (patient 2); arterial hypertension and hypercholesterolemia (patient 3); autoimmune disease with evidence of serum antimitochondrial antibodies and a tendency to arterial hypotension (patient 5); prostate cancer, anemia, a coagulation anomaly and diabetes mellitus (patient 6); hyperhomocysteinemia, hyperuricemia and a suspected diastolic disturbance of cardiac relaxation (patient 7); bronchopneumonia and a bilateral, medium-sized carotid stenosis (patient 8); heterozygous factor V mutation associated with venous thrombosis of the leg and arterial hypertension (patients 9 and 10); chronic smoking habit and a patent foramen ovale (patient 12); chronic smoker and a coagulation disorder (patient 13).

The three remaining (nos. 4, 11 and 14) presented less serious comorbidities: patient 4 had slightly elevated blood pressure and slightly increased homocysteine, patient 11 suffered from extremely high blood pressure, and patient 14 (who had an occlusion of a large cilioretinal artery) suffered from arterial hypertension. We identified no additional risk factors in these three patients.

Six patients presented coagulation anomalies in conjunction with other cardiovascular diseases, for instance the patient with paraneoplastic syndrome and diabetes mellitus. Arterial hypertension was diagnosed in 8 of 14 patients. One patient (no. 12) revealed a patent foramen ovale and hypertension. We detected no emboli in any of the patients ophthalmoscopically.

## Discussion

Concerning the risk profile, the cardiovascular risk factors for retinal artery occlusion are arterial hypertension, carotid artery diseases, cardiac rhythm disorders and cardiac valvular diseases, diabetes mellitus, hyperlipidemia, hyperuricemia and chronic smoking [3]. The cause of retinal artery occlusions in most patients is embolic. In our patients with combined CRAO with CRVO, however, no emboli were detected.

The main ophthalmoscopic feature of this entity is the absence of an embolus. Carotid artery examination and echocardiography in most of our patients failed to reveal cardiovascular anomalies that could lead to emboli. We carried out Doppler sonography in nine patients, of whom eight had normal carotid arteries. Only one patient revealed a moderate bilateral carotid stenosis.

Brown et al. [8] also reported the absence of carotid stenosis in most of their patients. Echocardiography was carried out in 11 patients in our group. Eight of them revealed no abnormalities, and only one patient, a chronic smoker, presented a patent foramen ovale (no. 12). Another patient revealed ventricular hypertrophy (no. 11), and a diastolic relaxation disturbance was suspected in an additional patient (no. 7).

The risk factors for retinal vein occlusion are systemic hypertension, diabetes mellitus and open-angle glaucoma [1]. In patients with combined CRAO with CRVO, however, the risk profile seems to differ because a combination of cardiovascular risk factors is essential in this entity. Eleven of our 14 patients presented comorbidities in the form of several cardiovascular risk factors.

The literature describes several systemic diseases, some of which were detected in our patients, that can cause this vascular entity, i.e., coagulation disturbances, arterial hypertension, arteriosclerosis, mechanical vessel compression at the optic nerve level, vascular inflammation, immunological diseases, systemic lupus erythematosus, Behçet’s disease, non-Hodgkin’s lymphoma and interferon therapy.

Comorbidities are also described in some patients in the literature. Vallée et al. [49] reported on 1 of 11 patients who suffered from hypertension, anticardiolipin syndrome and von Willebrand syndrome. Another patient revealed hypercholesterolemia and protein S deficiency. In an additional patient who smoked, hypertension and monoclonal gammopathy were diagnosed.

Glacet-Bernard et al. [17] described one of seven patients who was a chronic smoker and presented hypertension as well as hypercholesterolemia, hyperuricemia and hyperfibrinogenemia.

A 62-year-old patient suffered from prostate cancer and diabetes mellitus [25]. Leibovitch et al. [27] diagnosed systemic lupus erythematosus with lupus nephritis and severe hypertension in a 23-year-old man.

We assume that combined CRAO and CRVO occur simultaneously or within a short interval. However, an exception to this was published by Ang et al. [4]: a patient with an antiphospholipid syndrome initially revealed a CRVO and 1 month later an ophthalmic artery occlusion. Rachitskaya et al. [36] reported a CRVO in a 50-year-old man who presented a CRAO 2 weeks later.

Total bilateral blindness in conjunction with a complete arterial and venous interruption in blood flow was described by Coppeto & Lessell [12]. They reported severe bilateral retinal vasculitis with total retinal circulation arrest from thrombosis in most of the retinal vessels, including major arterioles, due to lupus erythematosus. That patient’s history also differed from our patients’ medical histories. Bilateral visual deterioration or blindness has also been reported [5,6,19].

We diagnosed a combined occlusion of a cilioretinal artery (CLRAO) with a CRVO in one patient (no. 14). Schatz et al. [41] observed ten patients presenting this combined occlusion. Schatz et al. noted pulsations in the cilioretinal artery in five eyes. They emphasized that the cilioretinal artery occlusion was not embolic. Due to lower perfusion pressure, the cilioretinal artery becomes relatively occluded. Their ten patients, all under 50 years of age, revealed cilioretinal artery occlusion associated with a non-ischemic CRVO. This disease’s prognosis is generally good. Eight of those nine eyes demonstrated a final visual acuity of 20/30 or better.

The prognosis of combined CRAO and CRVO, even if incomplete, is very poor, including blindness if left untreated. Such combined vascular retinal occlusions are caused by systemic diseases that should be treated at an early stage by an ophthalmologist and internist working together. As the systemic diseases in our patients are fundamental, treatment should be carried out in cooperation with internists.

Vallée et al. [49] are the only authors who treated patients with combined CRAO and CRVO via fibrinolysis. They selectively perfused urokinase via the femoral artery into the ophthalmic artery. Seven of eleven of their patients’ vision improved substantially, while one revealed an intravitreal hemorrhage with visual deterioration.

Patients with a combined retinal arteriovenous occlusion should be carefully followed up over months or years because of threatening neovascularization glaucoma. Rubeosis iridis was observed by Brown et al. [8], Smith [44], Stowe et al. [45], Richards [37], Sullivan et al. [46], Duker et al. [13] and Rachitskaya et al. [36].

Brown et al. [8] observed rubeosis iridis in 17 out of 21 patients (81%) with at least 6-month follow-up.

## Conclusions

The essential findings in our 14 patients were:

1) Unilateral simultaneous combined incomplete retinal artery and vein occlusions should be considered as one entity,

2) Eleven patients (78.6%) revealed co-morbidities presenting as cardiovascular risk factors such as arterial hypertension, hypercholesterolemia and chronic smoking. Immunological diseases, malignancies and coagulopathies may lead to this ocular disorder, causing blindness.

3) Normal echocardiography in eight patients and normal Doppler sonography findings in nine patients were revealed.

4) The mean age was 57.6 years (33–75).

5) None of our patients presented an embolism. This is therefore the main difference from an isolated CRAO or BRAO, as they often are caused by embolic events.

6) Patients suffering from acute visual loss must be examined for the presence of a systemic disease to enable treatment at an early stage.

## Consent

Written informed consent was obtained from the patient for the publication of this report and any accompanying images.

## Competing interests

The author declared that he has no competing interests.
